# Structure and Antimicrobial Properties of Monensin A and Its Derivatives: Summary of the Achievements

**DOI:** 10.1155/2013/742149

**Published:** 2013-02-13

**Authors:** Daniel Łowicki, Adam Huczyński

**Affiliations:** ^1^Faculty of Chemistry, Jagiellonian University, Ingardena 3, 30-060 Krakow, Poland; ^2^Department of Biochemistry, Faculty of Chemistry, Adam Mickiewicz University, Umultowska 89b, 61-614 Poznan, Poland

## Abstract

In this paper structural and microbiological studies on the ionophorous antibiotic monensin A and its derivatives have been collected. Monensin A is an ionophore which selectively complexes and transports sodium cation across lipid membranes, and therefore it shows a variety of biological properties. This antibiotic is commonly used as coccidiostat and nonhormonal growth promoter. The paper focuses on both the latest and earlier achievements concerning monensin A antimicrobial activity. The activities of monensin derivatives, including modifications of hydroxyl groups and carboxyl group, are also presented.

## 1. Introduction

Ionophores are the class of compounds making complexes with cation and then transporting it as lipid-soluble complex across lipid bilayer. MonensinA is a representative of a large group of naturally occurring polyether ionophorous antibiotics. It was discovered in 1967 by Agtarap etal. [1] as a metabolite formed in a biosynthesis of *Streptomyces cinnamonensis* bacteria. The details of monensin isolation are given in a separate work [2]. The appropriate mechanism of action of ionophores has been presented by Pressman et al. [3] in 1967, which marks this date as the beginning of the chemistry of ionophores. Elucidation of the crystal structure of monensin silver salt complex by Agtarap etal. [1], as well as that of a crystal complex of another previously known ionophore-nonactin with potassium cation by Kilbourn etal. [4], has significantly contributed to the understanding of this mechanism. Since that time a huge number of ionophorous antibiotics have been discovered, and their structures and antimicrobial properties have been studied. The group of carboxylic ionophores currently consists of nearly a hundred of compounds. However, only a few of them have been approved for use in veterinary practice. 

## 2. Structure of Monensin A and Its Complexes

### 2.1. Studies on the Monensin A Structure

**Scheme 1:**
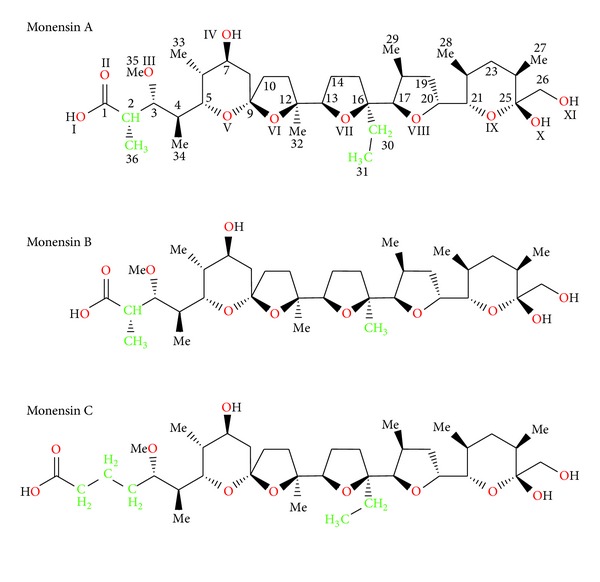
The formula and atom numbering of monensinA and its homologs.

**Figure 1:**
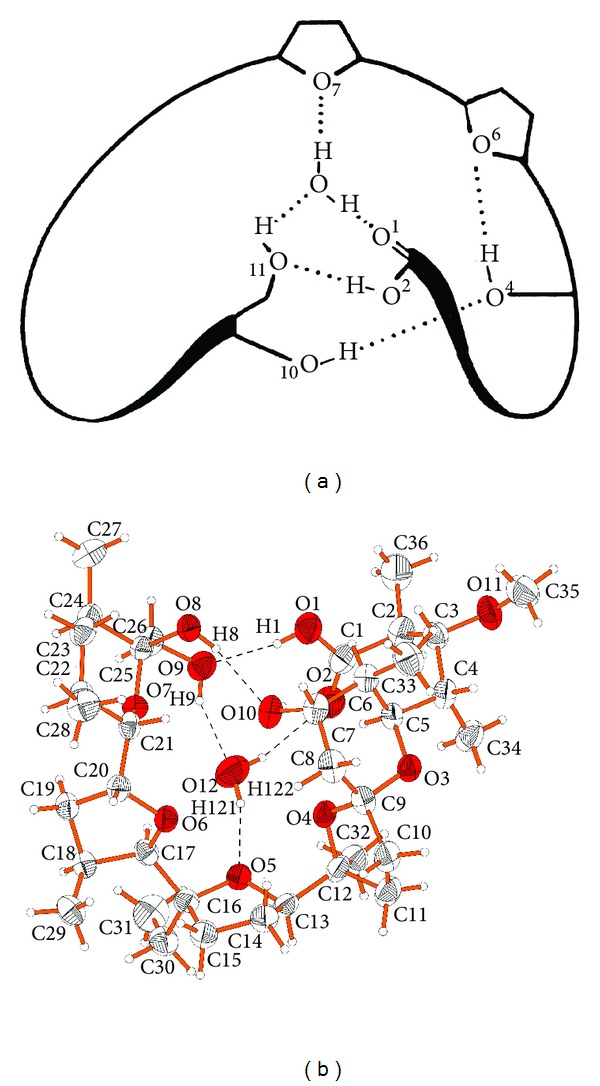
Structure of MONA monohydrate: (a) schematic representation [5], (b) complete crystal structure [6].

Monensin A, (Scheme 1) also called monensic acid, MONA, or MonH, occurs as a monohydrate with a water molecule complexed inside. The molecule of this ionophore contains six oxygen atoms, five of which may participate in the complexation of cations. Monensin molecule is maintained in a pseudocyclic conformation due to the presence of bifurcated intramolecular hydrogen bonds formed between carboxyl group on one side of the molecule and two hydroxyl groups on the opposite side. For the first time, the crystal structure of monensic acid monohydrate has been presented by Lutz etal. in 1971 [5]. The host-guest system of C_36_H_62_O_11_-H_2_O formula is stabilised by inter- and intramolecular hydrogen bonds (Figure 1(a)). This complex crystallises from absolute ethanol in orthorhombic crystal system and *P*2_1_2_1_2_1_ space group.

Recently, Huczyński etal. have presented the crystal structure of monensinA monohydrate obtained by crystallisation from acetonitrile. In comparison to Lutz's studies, the new structure contains all hydrogen atoms (Figure 1(b)) [6]. Until now, no crystal form of monensic acid without water has been presented, which could suggest that the anhydrous monensin cannot exist. Huczyński and coauthors demonstrated that in the dichloromethane solution (DCM) monensinA also exists as monohydrate, which has been proved by FTIR and NMR spectroscopies. 

A total synthesis of monensin was undertaken by Kishi et al. in 1979 [15–17]. This achievement is one of the first examples of stereoselective total synthesis through acyclic stereocontrol. Barely a year later Still and coworkers [18–20] presented another method for the synthesis of monensin.

### 2.2. Studies of the Monensin A Complexes

**Figure 2:**
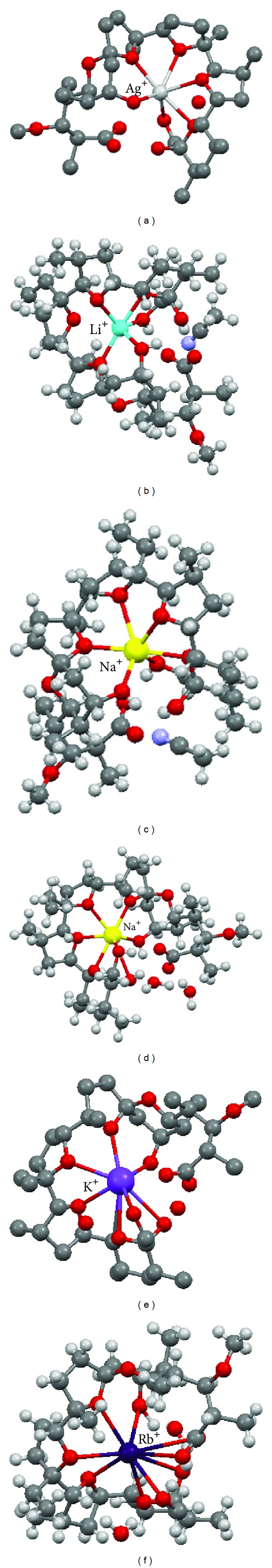
Crystal structures of monensin salts with (a) Ag^+^ dihydrate [14], (b and c) Li^+^ [7], and Na^+^ [8] inclusion complexes with ACN molecule, respectively, (d) Na^+^ [9], (e) K^+^ [11], and (f) Rb^+^ [12] dihydrates.

Ionophoretic properties of monensin A were widely studied in the last century [21–26]. Stability constants of monensin complexes with monovalent metal cations such as Li^+^, Na^+^, K^+^, Rb^+^, Cs^+^, and Ag^+^ were defined by various methods including fluorimetry, calorimetry, relaxation, electrochemical, and ^1^H NMR measurements. Determination of p*K*_*a*_ values was conducted in various protic, as well as polar aprotic solvents, and at different temperatures. Popov and coworkers have noted that monensin can form different types of complexes in solution, that is, monensin metal salt called monensinates of Mon^−^M^+^ formula and monensic acid with inorganic salts of MonH-M^+^X^−^ formula [21, 22]. Complexation titrations with other cations have shown that the selectivity of monensin varies in the order Ag^+^ > Na^+^ > K^+^ > Rb^+^ > Cs^+^ > Li^+^≈NH_4_^+^ [21]. Lutz etal. [27] have demonstrated that the ability to form complexes with monovalent cations by monensin is as follows: Na^+^ > K^+^ > Li^+^ > Rb^+^ > Cs^+^. A number of crystal salts of monensin A with different cations have been synthesized, and their structures were studied by X-ray diffraction (see Table 1 and Figure 2) [7–14]. The structure of sodium monensinate in chloroform solution was studied by Turner. The author applied nuclear Overhauser effect spectroscopy to study conformation of this complex [28]. Full assignment of ^13^C and ^1^H NMR signals of MONA-NaCl and MONA-NaClO_4_ complexes in DCM solution was performed by Huczyński etal. 

**Figure 3:**
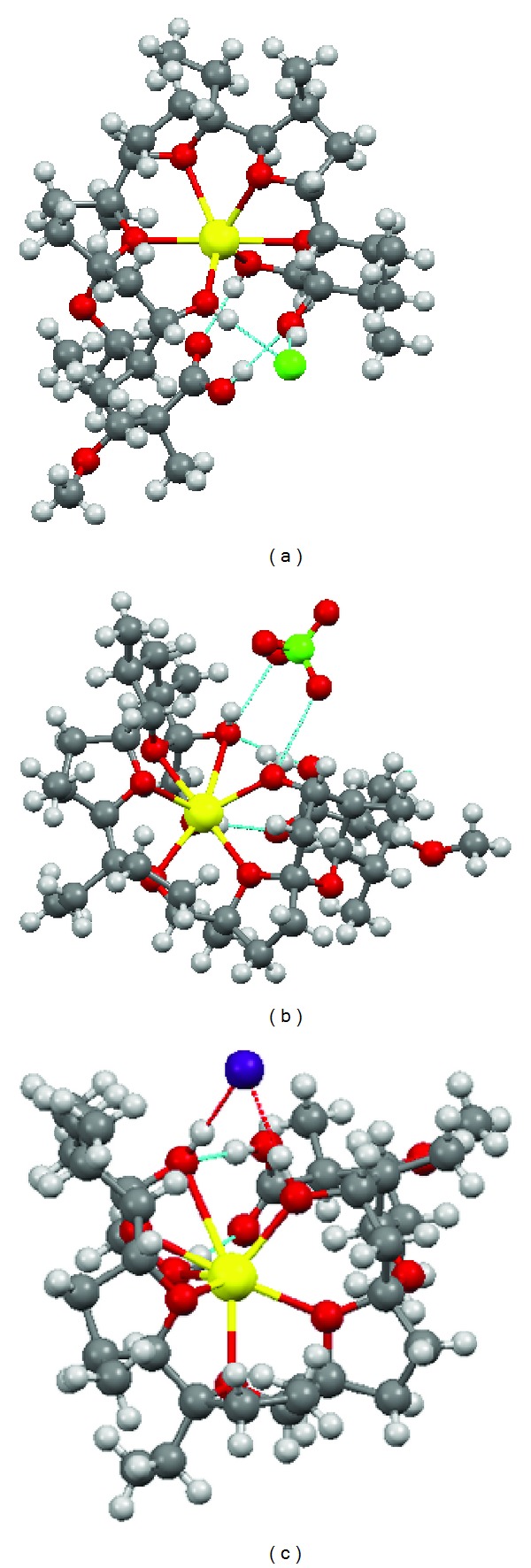
Crystal structures of monensin A free acid complexes with (a) NaCl, (b) NaClO_4_, and (c) NaBr [6, 29].

Three crystal complexes of monensin A with NaBr [29], NaCl, and NaClO_4_ have been synthesised [6] (Figure 3). Przybylski and coauthors calculated the structures of monensin A sodium, potassium and silver salts, monensin-free acid monohydrate, and some monensin derivative complexes. The semiempirical calculations were carried out by AM1, PM3, and PM5 parametric methods. Comparison of the calculated parameters (bond lengths and angles) of these structures with those in crystals has shown that the best results are achieved by PM5 method [30]. 

**Figure 4:**
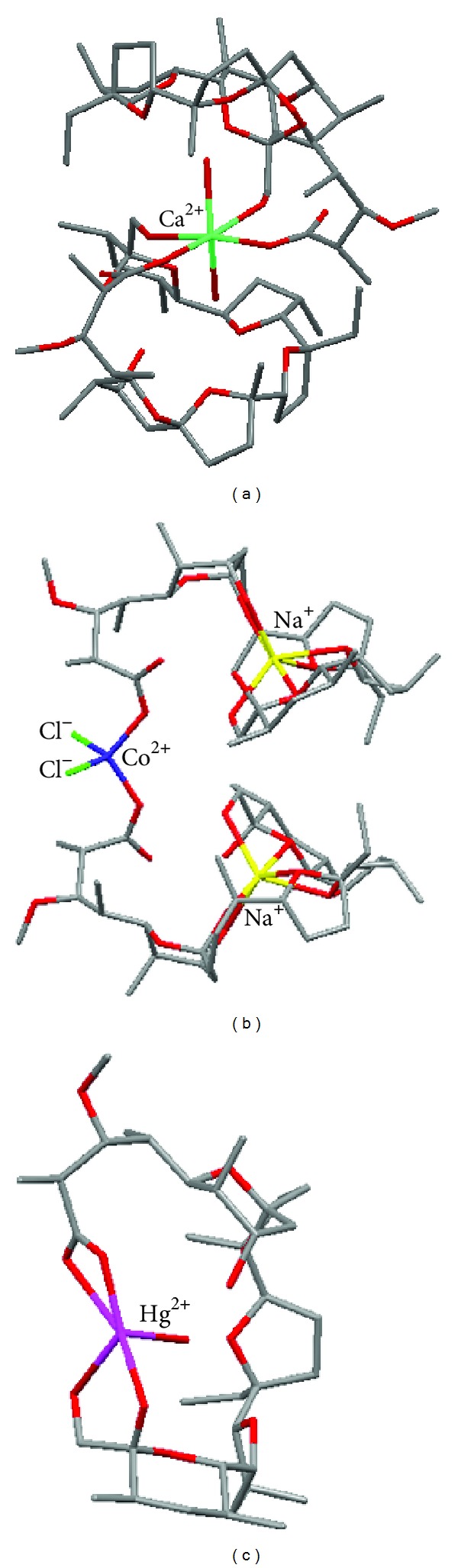
Crystal structures of (a) Ca(Mon)_2_(H_2_O)_2_, (b) Co(Mon^−^Na^+^)_2_Cl_2_, and (c) HgMon-H_2_O (protons are omitted for clarity).

Recently, crystal complexes of monensin with some divalent metal cations have been synthesised by Pantcheva and coworkers [31–36]. They found that three different types of complexes can be formed, but the divalent cation is not placed in hydrophilic cavity of the ionophore in any case. The first type of complexes is formed with the cations such as Mg^2+^, Ca^2+^, Zn^2+^, Cd^2+^, Co^2+^, Mn^2+^, and Ni^2+^. These complexes of [M(Mon)_2_(H_2_O)_2_] formula, where M is divalent metal, are neutral salts with two monensinates anions bound in a bidentate coordination mode to the cation. The metal cation is additionally coordinated by two water molecules placed in axial positions of the octahedron. The crystal structure of the [Ca(Mon)_2_(H_2_O)_2_] complex, which is a representative of this type complexes is shown in Figure 4(a) [31–34]. In the second type of complexes with such cations as Co^2+^, Mn^2+^, and Cu^2+^, two molecules of monensin sodium salt are bound with a divalent metal cation, which is simultaneously bound with two chloride anions Figure 4(b) [35, 36]. The complex with Hg(II) has quite a different structure in which one cation is bound with only one molecule of monensin (Figure 4(c)). The cation is located between carboxyl anion and two hydroxyl groups. The twofold negative charge of the ligand is achieved by deprotonation of carboxylic group and secondary hydroxyl group [34].

## 3. Biosynthesis of Monensin A

As already mentioned, monensin was isolated for the first time in 1967. Several homologues of it are known (Scheme 1), but the most famous is monensin A. The isolation process involves the biosynthesis and extraction of the monensin sodium salt in the culture of *Streptomyces cinnamonensis* actinobacteria, which is carried out in a complex medium containing glucose, soybean oil, and grit. Cultivation process is carried out for about a week at 30 °C and under intensive aeration [37–39]. Biosynthesis of monensin proceeds through the polyketide pathway with the biochemical processes similar to those taking place in fatty acid biosynthesis. The precursors are propionyl-CoA and malonyl-CoA, which provide acethyl, propionyl, and butyrate units. During the biosynthesis the following processes occur:

- binding of acyl groups,
- condensation of next malonyl-CoA molecule with emission of CO_2_,
- reduction of ketone group,
- emission of water molecule,
- reduction of the double bond.

In the biosynthesis performed with acetyl-CoA and malonyl-CoA, a multienzymatic protein complex is involved, which plays an important role of an acyl residue carrier. The information on the biosynthesis of monensin was obtained through research with the use of isotopes ^18^O- and ^13^C-labelled molecules [37]. The product of biosynthesis is excreted from the bacterial cells, and its concentration in the culture averages a few grams per litre. After completion of the biosynthesis process, the solid components are filtered off, and the filtrate is acidified to pH 3 and then extracted with chloroform. The extract is purified on activated carbon, concentrated, and crystallised. If the monensin concentration in a crude biosynthesis product is higher than 10 g/L, extraction with n-hexanol is carried out directly from this mixture. Subsequently, a water is removed by azeotropic distillation, and product is crystallised [37]. 

## 4. Properties and Toxicity of Monensin A

Since its discovery, monensin A has become an object of scientific interest because of its biological and pharmacological properties. Brief characterisation and toxicity of monensin A are presented in Table 2. At first its cellular effects on the Golgi apparatus, both in plant and animal cells, were understood. Monensin inhibits growth of selected cells by blocking the intracellular transport of the Golgi apparatus proteins, with no apparent inhibition of the synthesis of these proteins. Also, the transfer of products formed within the Golgi structures is inhibited by monensin [20, 40].

When exposed to monensin, the culture of plant cells slows down their growth or selected cellular processes, and usually the changes in functioning and structure of the Golgi apparatus occur. However, in the animal cells monensin induces mitochondrial damage without apparent change in the operation of the Golgi structure [43]. Monensin also slows down and reduces the process of endocytosis, that is, transport of large molecules through the cell membrane with the participation of a peptide transporter [44, 45]. The antibiotic induces pH change within the cellular structure, which can lead to a reduction in the secretion and/or transportation of the chemicals important for the proper functioning of the cell. Monensin also affects the processes of formation of external structures on the cell surface and their growth, by reducing the secretion of substances responsible for these processes (i.e., proteoglycans, collagen and procollagen, and fibronectin) [43]. Cellular effects of monensin depend on the body subjected to its action, the route of administration, and the dose of this antibiotic.

The main cellular effects caused by monensin are listed in Table 3. The very few studies performed on the antitumor activity of monensin have demonstrated that it can inhibit the proliferation of renal cancer cells by inducing apoptosis in cancer cells and cell cycle arrest in G or G2-M phases [46].

## 5. Antimicrobial Activity of Monensin A

Monensin is one of the most widely studied ionophore antibiotics. Most of the work has been devoted to its biological activity, including the antimicrobial properties. Monensin antibacterial activity can be explained by changes in pH and the sodium-potassium balance in the cell, which leads to critical disturbances in cellular processes, resulting in cell death [47]. Monensin and some of its derivatives have shown activity against Gram-positive bacteria of the genera *Micrococcus*, *Bacillus,* and *Staphylococcus* [35, 48, 49]. It has been found that only Gram-positive G(+) bacteria are sensitive to monensin, which may be due to the fact that the cell walls of Gram-negative G(−) bacteria have more complexed construction not permeable to large antibiotic molecules and the complexes formed by it. The studies devoted to the antiviral properties of monensin indicate that it is active against some viruses. Inhibition of vesicular stomatitis and Sindbis virus replication have been demonstrated [50, 51]. Monensin has been also found to inhibit Semliki Forest virus penetration into the target cells [45]. Iacoangeli and coworkers have shown in their studies that monensin decreases DNA synthesis, effectively inhibits the replication, and induces a strong reduction of early viral antigens of murine polyoma virus [52]. *In vitro* studies of monensin showed pronounced activity against *Plasmodium falciparum*, much stronger than that of the antimalarial drug—chloroquine. In clinical *in vivo* studies in mice infected with *Plasmodium vinckei petteri*, a 100% animals have been cured after treatment with monensin doses of 10 mg/kg. Antimalarial action of monensin can be explained by impaired function of nutrient and other vacuolar organelles of the parasite and the intracellular acidification, which eventually lead to the cell death [53].

## 6. Ion Transport

**Figure 5:**
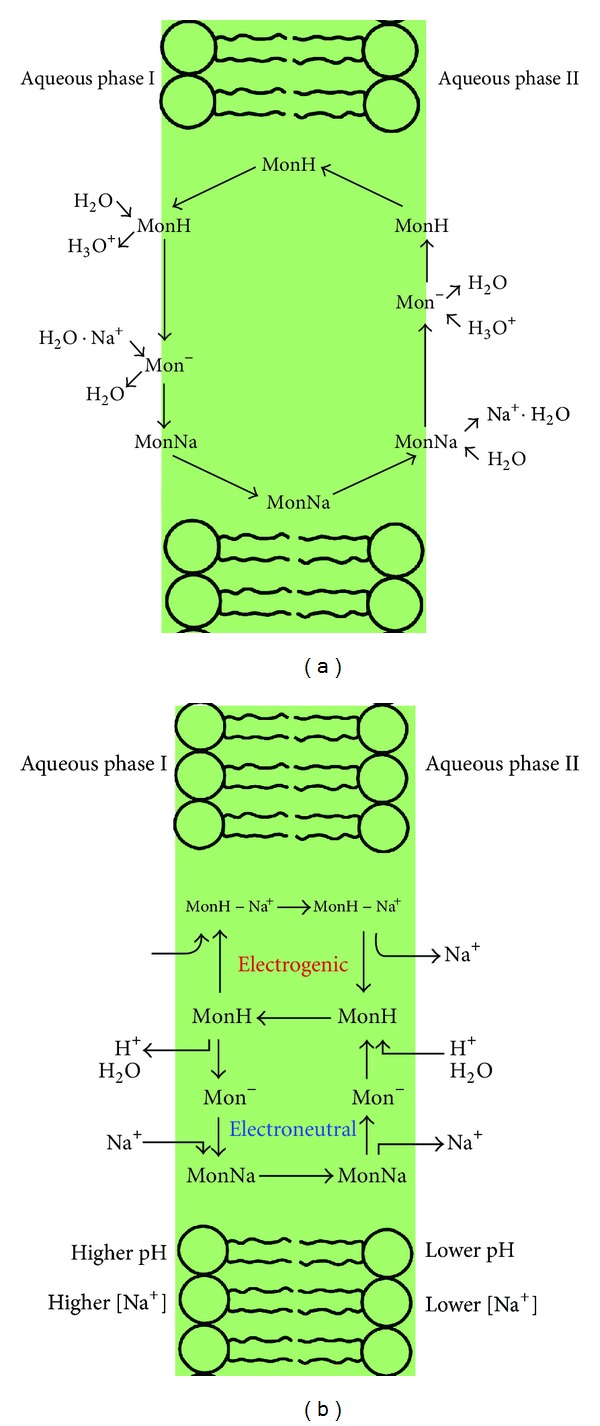
Different models of ion transport by monensin: (a) electroneutral, (b) mixed electroneutral and electrogenic.

It has long been known that the biological activity of monensin arises from its ability to complex with the sodium cation and transport it across cell membranes. Initially it was thought that the mechanism of ion transport is a simple antiport of Na^+^/H^+^ cations [54–56]. The hypothesis assumes that monensin molecule binds the sodium cation as a salt, loosing proton from carboxyl group, and moves the cation as a complex on the opposite side of the lipid membrane (Figure 5(a)). Then, the sodium cation is released, and carboxylate anion undergoes protonation.

A neutral acid molecule migrates back to the other side of the membrane. This process is powered by difference of cation concentrations inside and outside the cell and tends to align the ions gradient. This mechanism of transport is electroneutral. However, microbiological studies of monensin A derivatives with blocked carboxyl groups such as amides and esters have also shown their antimicrobial properties. In 1991 Nakazato and Hatano have measured the fluxes of Na^+^ and H^+^ using monensin A containing liposomes and concluded that Na^+^ is transported in the form of a 1 : 1 complex between monensic acid (MonH) and Na^+^ cation [57]. Recently, Huczyński etal. have synthesised two complexes of monensin acid with NaCl and NaClO_4_ and determined their crystal structures. The authors have proven by FT-IR study that the structure of the complexes is conserved in the hydrophobic environment like in DCM solution [6]. These findings support the assumption that the [MonH·M^+^X^−^] type complexes can exist in hydrophobic membranes and affect the Na^+^ transport also in electrogenic way (Figure 5(b)).

## 7. Applications of Monensin A

There are over 100 known ionophore antibiotics, but only three, monensin, salinomycin, and lasalocid acid, have found currently commercial application. Monensin was the first ionophoric antibiotic approved for use by the Food and Drug Administration (FDA) in the USA. Monensin A is an antibiotic which is used as coccidiostat and growth promoting agent in veterinary practice. Due to the strong antibacterial and coccidiostatic properties it has found application in industrial poultry farming. Coccidia are parasitic protozoa commonly occurring in different animal species, which multiply in the intestinal epithelial cells and propagate through the oocysts excreted in the faeces. These microorganisms cause inflammation of the mucous membrane of the small intestine, resulting in diarrheal and general weakness. The mechanism of coccidiostatic activity of monensin involves blocking the development of trophozoites of protozoa in the *Eimeria* genus of *Coccicdium* group, in the first phase of schizogony. As a result, a positive effect of antibiotic on the growth of poultry is to reduce the proliferation of parasites, thus eliminating attenuation on stockbreeding [58, 59]. For the prevention of coccidiosis in poultry Mondolar formulation, containing 10% or 20% sodium monensin, is used. Subsequent studies have shown that monensin may also improve food metabolism in ruminants, which ensures its better use and, consequently, leads to faster growth of cattle. Stimulation of growth is associated in this case with favourable changes of intestinal bovine microflora and increasing amounts of assimilable digested protein. Rumenesin containing 6.6% of monensin is used as a nonhormonal growth promoter for animals [27, 60, 61]. Monensin used as a coccidiostat in poultry or growth promoter in cattle is relatively safe if used in the recommended doses. However, the possibility of poisoning animals as well as antibiotic contamination of animal products (meat, eggs, and milk) must always be taken into account. Due to a broad spectrum of biological activity, monensin derivatives are an important object of research aimed at reducing the toxicity and to obtain new compounds with improved biological properties in terms of further use.

## 8. Monensin A Derivatives and Their Antimicrobial Activity

A broad spectrum of antimicrobial and biological properties of monensin has made the chemical modification of monensin a very interesting direction of research. The novel derivatives of the ionophore, depending on the location of chemical modification, differ from the parent molecule in complexation selectivity, structure of complexes formed, ion transport mechanism, and toxicity and biological properties, including antimicrobial activity. Several research groups have synthesized a wide range of monensin derivatives, some of which have been tested for antimicrobial activity. 

### 8.1. Modifications of Hydroxyl Groups

**Figure 6:**
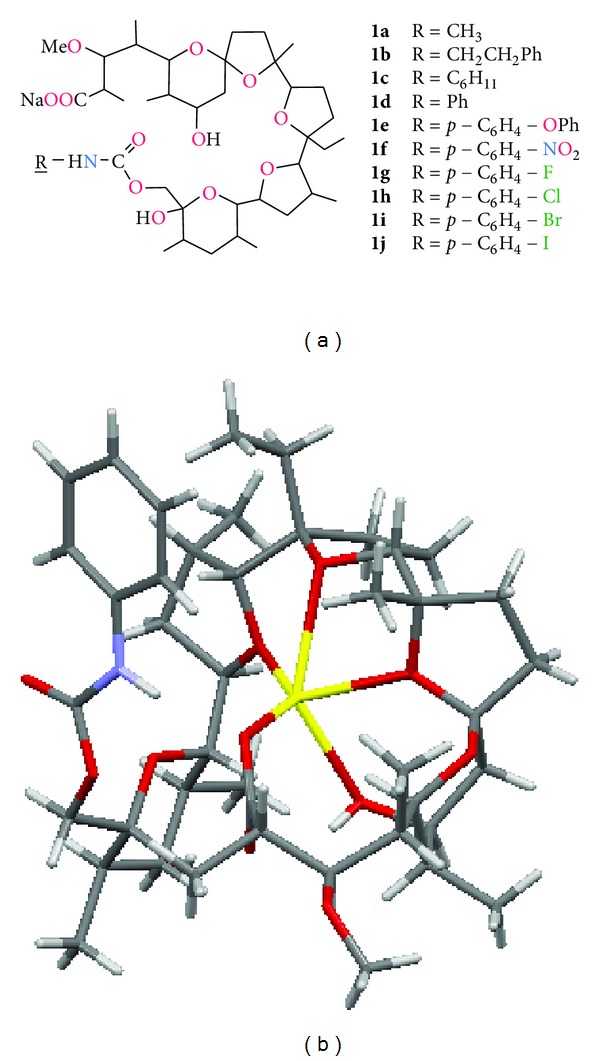
Structures of selected monensin A urethanes (a) [41]; crystal structure of monensin sodium urethane (b) [42].

Chemical modification of all three hydroxyl groups has already been carried out. Westley and coworkers have reported the synthesis and antimicrobial properties of a series of monensin urethanes, obtained by modification of O(XI)H group (Figure 6, compounds **1a–j**) [41]. These derivatives are very interesting in terms of chemical and microbiological properties, because they are able to transport monovalent cations about 10 times more effectively than monensin. The urethanes also show up to 10-fold higher activity compared to unmodified monensin against G(+) bacteria (MIC values varied from 0.02 to over 25 *μ*g/mL). Moreover, some of these compounds are also active against *Candida albicans* fungus (MIC = 0.08 *μ*g/mL to more than 100 *μ*g/mL for urethane, MIC > 100 *μ*g/mL for unmodified MONA) and *Penicillium digitatum* (MIC 6.3 *μ*g/mL to more than 100 *μ*g/mL for urethanes, MIC > 100 *μ*g/mL for unmodified monensin). 

**Figure 7:**
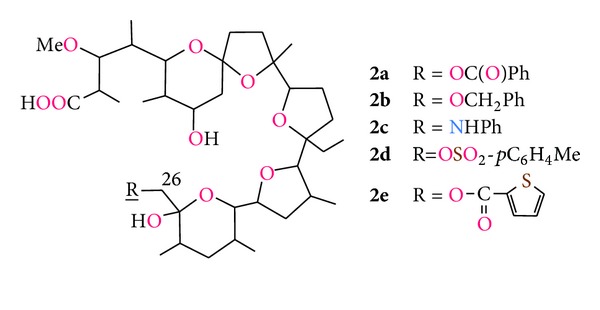
Monensin A derivatives modified at C(26) atom.

Furthermore, four of the monensin urethanes obtained showed antimalarial properties in *in vivo* tests [41]. Westley et al. have postulated that in the molecular structure of sodium monensin urethanes the oxygen of urethane carbonyl group coordinates metal cation. Recently Huczyński etal. [42] reinvestigated the structure of the phenyl urethane of MonNa complex and have shown that in both, solution and crystal structure, this group does not participate in the process of complexation (Figure 6). Chemical modification of monensin at the C(26) carbon atom was also carried out to prepare various derivatives of the antibiotic including: esters, ethers, amine, and sulphonate (Figure 7, compounds **2a–e**). Among all of these derivatives 26-fenylaminomonensin (**2c**) exhibits antimicrobial activity against various bacterial strains with MIC values of 0.20–6.25 *μ*g/mL. The activity of this compound was higher than that of monensin and even a very active derivative—26-phenylurethane of monensin (**1d**) [62]. The chemical modification of monensin at position C-26 can also lead to changes in preferences of cation complexation by this ionophore. Rochdi etal. [63] have demonstrated that **2d** and **2e** monensin derivatives cause an increase in the effective transport of potassium cation through the membrane, whereas there is a decrease in the transport of sodium cations. Thus, these derivatives preferentially complex and transport potassium cations prior to sodium cations. Such inversion of complexation selectivity by the chemical modification of monensin has also led to improvement of its antibacterial activity against *Bacillus cereus* as well as *in vivo* antimalarial activity toward *Plasmodium falciparum* [63]. 

**Figure 8:**
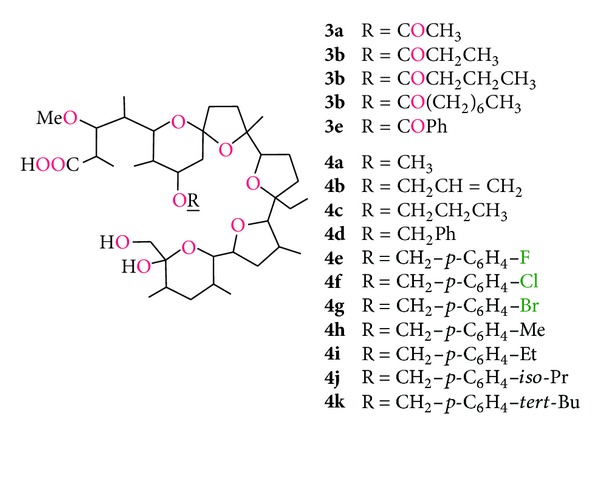
Monensin A derivatives with O(IV)H group modified.

Among monensin derivatives with modified O(IV)H hydroxyl group the ester derivatives (Figure 8, compounds **3a–e**) and ether derivatives (Figure 8, compounds **4a–k**) have been synthesized [64, 65]. Acyl derivatives of monensin **3a–e** showed lower antibacterial activity against both aerobic and anaerobic bacteria than the starting material. However, monensin-O(4) benzyl ethers (**4d–k**) showed much higher antibacterial activity than monensin. This can be related to the hydrophobic nature of the benzyl substituent, whose role is to improve the solubility of the derivatives of monensin in the bacterial cell membrane [65].

### 8.2. Modifications of Carboxyl Group

**Figure 9:**
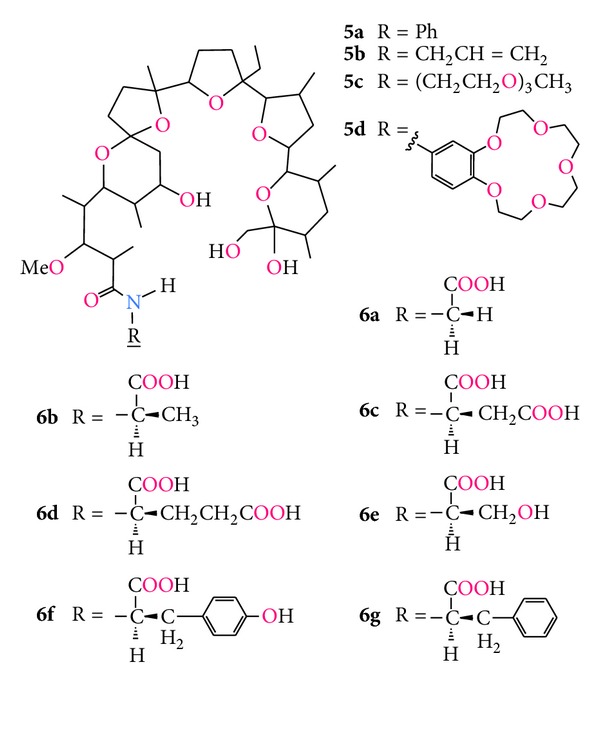
Structures of monensin amides.

Modifications of the carboxyl group include the synthesis of amides and esters of monensin A, most of which were performed by Professor Brzezinski's group. Recently, four new amides of monensin have been synthesised by Łowicki etal. [66–70], and their ability to complex monovalent metal cations such as Li^+^, Na^+^ and K^+^ has been studied (Figure 9, compounds **5a–d**). The reason why polyether antibiotics exhibit several pharmacological and biological effects is their ability to form lipid-soluble pseudocyclic complexes with metal cations and transport them through cell membranes disturbing their natural Na^+^/ K^+^ ion balance. Thus, studies of the biological activity of monensin derivatives should be always connected with the studies of their ionophoretic properties. 

**Figure 10:**
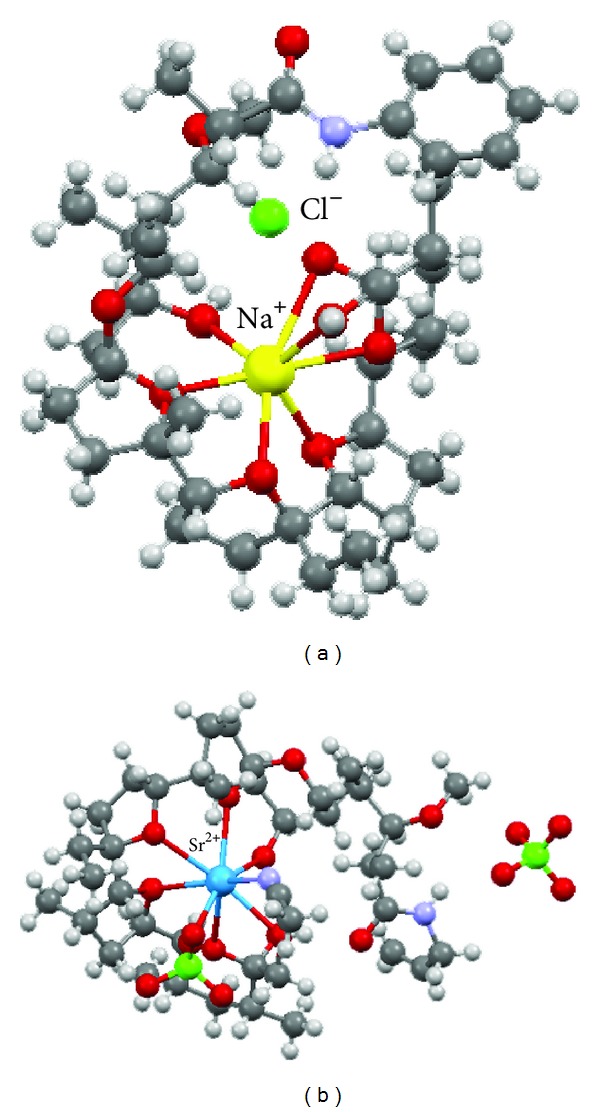
Crystal structures of monensin A amide complexes: (a) [**5a**—NaCl], (b) [**5b**—Sr(ClO_4_)_2_-CH_3_CN].

Among all the amide complexes studied in solution, only a complex of *N*-phenylamide with sodium chloride showed a tendency to crystallize, therefore its structure has been determined by crystallographic methods (Figure 10(a)) [66]. Moreover, **5b** and **5c** amides have been found to be able to bind alkaline earth metal cations such as Mg^2+^, Ca^2+^, Sr^2+^, and Ba^2+^ [71]. Surprisingly, the complex of **5b** with strontium perchlorate crystallized from acetonitrile solution and its structure has been determined (Figure 10(b)) [72]. It is worth noting that the Sr^2+^ cation is placed inside the hydrophilic cavity of monensin amide molecule, which is the first example of that kind of complex with divalent metal cation. The ability to complex sodium cations by two complexation centres in monensin amide with 4-aminobenzo-15-crown-5 was investigated by spectroscopic and spectrometric methods. These studies have proved that the Na^+^ cation in 1 : 1 complex is bound in the monensin part of **5d** molecule; however, the complex of 1 : 2 stoichiometry can also be formed [70]. All the monensin amides were tested *in vitro* toward G(+), G(−) bacteria and yeasts, and they showed comparable, but a little bit smaller activity, against G(+) cocci than that of the parent ionophore (see Table 4).

The *N*-phenyl amide (**5a**) gave the best results in the *in vitro* tests on standard strains, thus it was additionally subjected to studies toward some strains of methicillin-resistant, methicillin-susceptible *S. aureus* (MRSA and MSSA), and methicillin-resistant *S. epidermidis* (MRSE). The **5a** amid showed significant activity against all tested hospital strains of MRSA, MSSA, and MRSE of MIC values between 6.25 and 25 *μ*g/mL [67]. 

**Figure 11:**
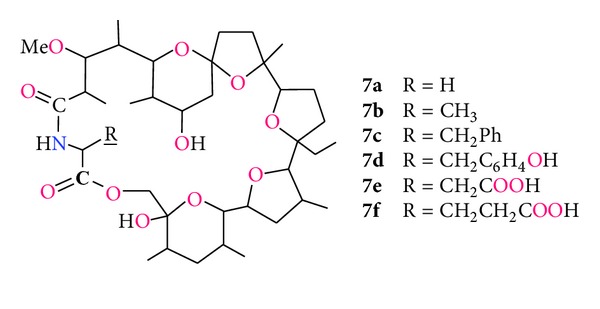
Structures of monensylamino acid lactones.

In 1988 Japanese research group led by Sakakibara synthesised several amides of monensin A with chiral amino acids (Figure 9, compounds **6a–g**). Subsequently, they synthesised crystal complexes of these amides with sodium bromide [73]. Further modification of the monensylamino acids included the lactamization reaction in which the carboxyl group of the corresponding amino acid moiety is connected with monensin O(XI)H hydroxyl group (Figure 11, compounds **7a–f**).

The macrocyclic lactones were tested for their activity against anaerobic bacteria and showed a lower efficiency than that of unmodified monensin A. For example, the values of the minimum inhibitory concentration of growth *Peptostreptococcus anaerobius* strain B-30 ranged from 25 to 50 *μ*g/mL for compounds **7a–f**, whereas the MIC value for the monensin was 1.56 *μ*g/mL [74].

**Figure 12:**
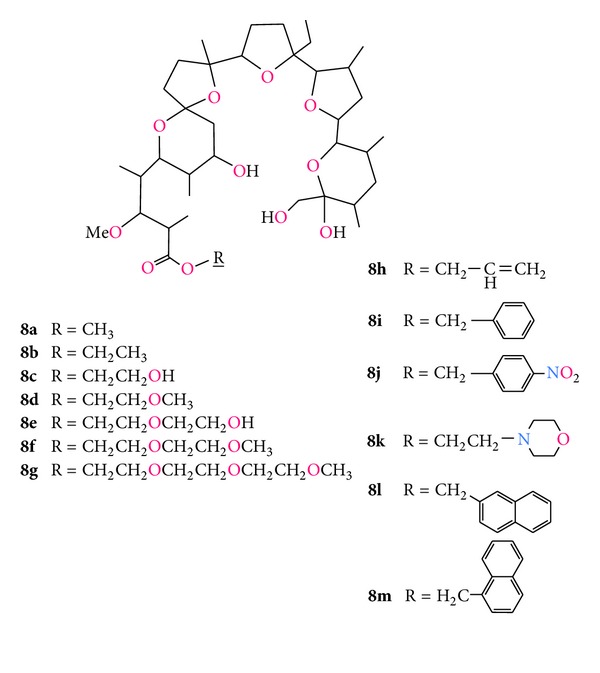
Structures of monensin A esters.

The largest group of monensin A derivatives are esters, most of which have been synthesised by Huczyński etal. during the last decade (Figure 12, compound **8a–m**). The ability of the esters to form complexes with monovalent alkali [75–82] as well as divalent alkaline earth metal cations [83–85] has been investigated. 

**Figure 13:**
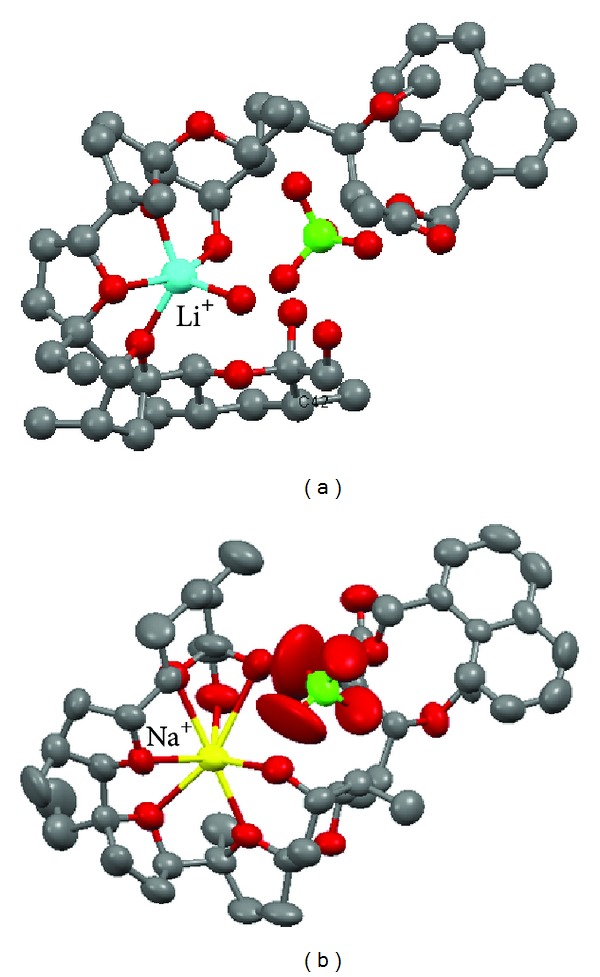
Crystal structures of (a) [**8m**—LiClO_4_-H_2_O] and (b) [**8m**—NaClO_4_] complexes.

Two complexes of monensin esters have been obtained in crystal form, that is, aqualithium 1-naphthylmethyl ester of monensin perchlorate (Figure 13(a)) [86], and 1-naphthylmethyl ester of monensin with sodium perchlorate (Figure 13(b)) [87]. 

**Figure 14:**
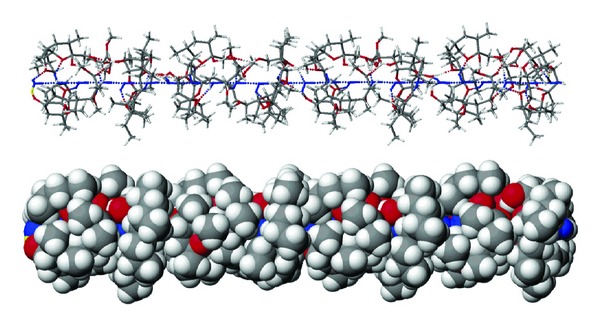
Ball and stick projection of the proton channel structure made up of eight (**8a** + 3H_2_O) species calculated by the PM5 method.

An interesting case is monensin methyl ester (**8a**) which is able to form a proton channel created by eight molecules of **8a** each with three water molecules bounded in the hydrophilic space. Within the channel all 24 water molecules are connected by almost linear hydrogen bonds (Figure 14) [88]. The H^+^ cation can be attached on one side of the channel, while the other proton is dissociated on the opposite side of the channel. These studies show that monensin methyl ester can be recognised as a channel forming ionophore, while unmodified monensin is considered to be the typical ion carrier.

Antimicrobial properties of all the esters **8a–m** have been tested towards G(+), G(−) bacteria and yeasts [49]. Among the wide range of these derivatives only three (**8h**, **8g**, and **8k**) have shown activity against some strains of Gram-positive bacteria.

Monensin A and its esters, which showed activity against the strains of Gram-positive cocci, were subjected to additional testing on hospital strains of *S*.*aureus*, including methicillin-susceptible (MSSA) and methicillin-resistant (MRSA) strains.

These studies have indicated that monensin and 2-morpholinoethyl ester (**8k**) show high activity against both types of *S*.*aureus*, that is, MRSA and MSSA. The other two esters (**8h** and **8g**) show moderate activity. The MIC values of the monensin esters are compared with that of unmodified monensin in Table 5. 

In the Professor Brzezinski's group the synthesis of macromolecular derivatives of monensin (dimers and trimers) has also been developed. These derivatives are completely inactive against Gram-negative bacteria, because the compounds with high-molar masses are unable to penetrate the membrane of these bacteria. However, these compounds showed moderate activity in the assays against Gram-positive bacteria, their MIC values for G(+) bacteria ranged from 6.25 *μ*g/mL to 200 *μ*g/mL [89].

## Figures and Tables

**Table 1:** Chemical formula of monensin salts obtained in a crystal form.

Number	Chemical formula	References
1	Li(C_36_H_61_O_11_) · CH_3_CN	[7]
2	Na(C_36_H_61_O_11_) · CH_3_CN	[8]
3	Na(C_36_H_61_O_11_) · 2H_2_O	[9, 10]
4	K(C_36_H_61_O_11_) · 2H_2_O	[11]
5	Rb(C_36_H_61_O_11_) · 2H_2_O	[12, 13]
6	Ag(C_36_H_61_O_11_)	[1]
7	Ag(C_36_H_61_O_11_) · 2H_2_O	[14]

**Table 2:** Characterisation and toxicity of monensin A.

Name	Monensin A, monensic acid
CAS name	2-[5-Ethyltetrahydro-5-[tetrahydro-3-methyl-5-[tetrahydro-6-hydroxy-6-(hydroxymethyl)-3,5-dimethyl-2H-pyran-2-yl]-2-furyl]-2-furyl]-9-hydroxy-*β*-methoxy-*α*,*γ*,2,8-tetramethyl-1,6-dioxaspiro[4.5] decane-7-butyric acid
Molecular weight	670.87 g/mol
Molecular formula	C_36_H_62_O_11_
Composition	C 64,45%; H 9,32%; O 26,23%
Melting point	103–105°C (monohydrate)
Specific rotation	[*α*]D = +47,7° (methanol)

Toxicity in animals (oral administration of the sodium salt) LD50:	
Monkey	>160 mg/kg
Rabbit	42 mg/kg
Rat	29 mg/kg
Cattle	26 mg/kg
Swine	17 mg/kg
Dog	>10 mg/kg

**Table 3:** Selected cellular effects of monensin.

*Decreased secretion:* proteoglycans, prolactin, albumin, transferrin, proinsulin polypeptides, *α*-amylase isoenzyme, different proteins, thyroxine-binding globulin, gonadotropin-binding globulin, acetyl cholinesterase, phytohemagglutinin, VLD lipoproteins, and glycoproteins of vesicular stomatitis virus	
*Increased secretion:* catecholamines type of biogenic amines, proteolytic cascade enzyme—cathepsin D	
*Damaging proteins transformation processes:* changes proalbumin into albumin	
*The deformation of oligosaccharides:* herpes simplex virus glycoprotein, coronaviruses, myeloperoxidase, and fibronectin	
*Inhibition of assimilation:* horseradish peroxidase, arylsulfatase, immunoglobulins, and *α*-2-macroglobulin	
*Inhibition of assimilated ligands dissociation:* asialoglycoproteins, asialoorosomucoid	
*Inhibition of ligands transfer:* epidermal growth factor, *β*-hexosaminidase, immunoglobulins, low-density lipoprotein, and proteoglycans to the lysosomes	
*Inhibition of acidification:* endosomes, lysosomes, and exosomes	
*Impact on the processes of external cellular structures creating by reducing the secretion:* proteoglycans, collagen and procollagen, fibronectin, and lamin	

**Table 4:** Minimal inhibitory concentration (MIC in *μ*g/mL) of monensin and its amides toward different G(+) microorganisms.

Tested strain	MonA	**5a**	**5b**	**5c**	**5d**
*S. aureus* NCTC 4163	2	12.5	50	400	50
*S. aureus* ATCC 25923	1	12.5	50	400	50
*S. aureus* ATCC 6538	2	12.5	50	400	50
*S. aureus *ATCC 29213	1	12.5	50	>400	50
*S. epidermidis* ATCC 12228	2	12.5	100	>400	50
*B. subtilis* ATCC 6633	1	6.25	50	400	50
*B. cereus *ATCC 11778	2	6.25	25	200	25
*E. hirae* ATCC 10541	12.5	>400	>400	>400	400
*M. luteus *ATCC 9341	4	6.25	50	400	50
*M. luteus *ATCC 10240	2	6.25	50	200	50

**Table 5:** Minimal inhibitory concentration of monensin and its esters toward different G(+) microorganisms.

Tested strain	MonH	**8k**	**8h**	**8g**
*S. aureus* NCTC 4163	2	100	100	12.5
*S. aureus* ATCC 25923	1	100	50	6.25
*S. aureus* ATCC 6538	2	100	100	12.5
*S. aureus *ATCC 29213	1	100	50	6.25
*S. epidermidis* ATCC 12228	2	100	100	12.5
*B. subtilis* ATCC 6633	1	12.5	25	6.25
*B. cereus *ATCC 11778	2	12.5	50	6.25
*E. hirae* ATCC 10541	12.5	>400	>400	50
*M. luteus *ATCC 9341	4	100	200	25
*M. luteus *ATCC 10240	2	50	50	12.5
*C. albicans* ATCC 10231	*ia *	*ia *	*ia *	200
*C. albicans *ATCC 90028	*ia *	*ia *	*ia *	200
*C. parapsilosis *ATCC 22019	*ia *	*ia *	*ia *	400

*ia*: inactive compound.
